# Integrating interdisciplinary methodologies for One Health: goat farm re-implicated as the probable source of an urban Q fever outbreak, the Netherlands, 2009

**DOI:** 10.1186/s12879-015-1083-9

**Published:** 2015-09-03

**Authors:** Georgia A. F. Ladbury, Jeroen P.G. Van Leuken, Arno Swart, Piet Vellema, Barbara Schimmer, Ronald Ter Schegget, Wim Van der Hoek

**Affiliations:** Centre for Infectious Disease Control, National Institute for Public Health and the Environment (RIVM), PO Box 1, , 3720 BA Bilthoven, The Netherlands; European Programme for Intervention Epidemiology Training (EPIET), European Centre for Disease Prevention and Control, Tomtebodavägen 11a, 171 83 Stockholm, Sweden; Institute for Risk Assessment Sciences (IRAS), Utrecht University, Domplein 29, 3512 JE Utrecht, The Netherlands; Department of Small Ruminant Health, Animal Health Service (GD), Arnsbergstraat 7, 7418 EZ Deventer, The Netherlands; Municipal Health Service Brabant-Zuidoost, Clausplein 10, 5611 XP Eindhoven, The Netherlands

**Keywords:** Q fever, Atmospheric dispersion model, *Coxiella burnetii*, Spatio-temporal analysis, Airborne

## Abstract

**Background:**

In spring 2008, a goat farm experiencing Q fever abortions (“Farm A”) was identified as the probable source of a human Q fever outbreak in a Dutch town. In 2009, a larger outbreak with 347 cases occurred in the town, despite no clinical Q fever being reported from any local farm.

**Methods:**

Our study aimed to identify the source of the 2009 outbreak by applying a combination of interdisciplinary methods, using data from several sources and sectors, to investigate seventeen farms in the area: namely, descriptive epidemiology of notified cases; collation of veterinary data regarding the seventeen farms; spatial attack rate and relative risk analyses; and GIS mapping of farms and smooth incidence of cases. We conducted further spatio-temporal analyses that integrated temporal data regarding date of onset with spatial data from an atmospheric dispersion model with the most highly suspected source at the centre.

**Results:**

Our analyses indicated that Farm A was again the most likely source of infection, with persons living within 1 km of the farm at a 46 times larger risk of being a case compared to those living within 5-10 km. The spatio-temporal analyses demonstrated that about 60 – 65 % of the cases could be explained by aerosol transmission from Farm A assuming emission from week 9; these explained cases lived significantly closer to the farm than the unexplained cases (p = 0.004). A visit to Farm A revealed that there had been no particular changes in management during the spring/summer of 2009, nor any animal health problems around the time of parturition or at any other time during the year.

**Conclusions:**

We conclude that the probable source of the 2009 outbreak was the same farm implicated in 2008, despite animal health indicators being absent. Veterinary and public health professionals should consider farms with past as well as current history of Q fever as potential sources of human outbreaks.

**Electronic supplementary material:**

The online version of this article (doi:10.1186/s12879-015-1083-9) contains supplementary material, which is available to authorized users.

## Background

Q fever is a zoonosis caused by the bacterium *Coxiella burnetii* and is present worldwide with the exception of New Zealand. Clinical disease in humans can range from an acute influenza-like illness to a chronic infection manifesting mainly as endocarditis or vascular infection [1], though 60 % of infections are asymptomatic. There is also evidence of a “post Q-fever fatigue syndrome” manifesting as chronic fatigue following acute infection [2]. The animal reservoir for Q fever is large as a wide range of mammals, birds, reptiles and fish can become infected with *C. burnetii*, but cattle and small ruminants (sheep and goats) are the most affected [1]. Clinical presentation of Q fever in domestic ruminants is most commonly associated with late abortions, stillbirth and delivery of weak offspring [3–5]. The most frequent route of infection for humans is from inhalation of aerosols contaminated by infected ruminants at slaughter or parturition [1]. Windborne spread of such aerosols can cause infections several kilometres from their origin [1, 6–9]. Furthermore, environmental contamination related to parturition or slaughter can last potentially from months to years, making inhalation of contaminated dust a risk [1].

Between 2007 and 2010, the Netherlands experienced the largest outbreak of Q fever ever reported, with over 4,000 notified cases [10, 11] and mainly associated with dairy goat farms [10–13]. Since that time, a number of methodologies have been developed and applied to investigate the epidemic. These methods incorporate traditional epidemiological approaches alongside Geographic Information Systems (GIS) [12, 13], mathematical modelling [14], and atmospheric dispersion models [15]; and use data from a range of sectors and sources, including animal, human, environmental and meteorological. In this sense, the Q-fever epidemic has offered a unique opportunity to develop interdisciplinary, “One-Health” methodologies to investigate airborne and zoonotic disease.

In the spring/summer of 2008, a Q fever outbreak occurred in an urban area (approximately 88,000 inhabitants) in the south of the Netherlands with 96 cases notified to the local Municipal Health Service (MHS), Brabant-Zuidoost. Through use of a Geographic Information System, this outbreak was subsequently linked to a commercial dairy goat farm (Farm A) that had experienced a Q fever-related abortion wave in the weeks preceding the human outbreak [12]. Persons living within 2 km of the farm had over a thirty times higher risk for Q fever than those living more than 5 km away, with risk declining with increasing distance from the farm. This spatial relationship, together with the fact that no other farms in the locality had reported Q-fever problems, supported the hypothesis that this single goat farm was the source of the human outbreak.

In 2009, an even larger human outbreak occurred in the town, with 347 cases notified to the same MHS between April and July. However, in this year no farm in the locality reported any clinical Q fever or related animal health problems. The reasons for the upsurge in human cases were unclear. Hypotheses included: increased reporting of Q fever owing to greater awareness of the disease amongst medical professionals and the community; the existence of a new source or sources of infection; or Farm A being again the source due to sub-clinical shedding or environmental contamination. Our study aimed to integrate the interdisciplinary methodologies and inter-sectoral data developed and gathered since the start of the national epidemic to identify the most likely origin of the 2009 outbreak in the area.

## Methods

### Case definition

We defined cases as residents of the Dutch Municipal Health Service region ‘Brabant-Zuidoost’ who were notified to the national surveillance system for infectious diseases as having Q fever with date of onset of between weeks 11 and 36 (9 March-6 September), 2009. In 2009, the notification criteria for Q fever were (1) fever, pneumonia or hepatitis, and (2) evidence of *C. burnetii* infection through a fourfold rise in IgG antibody titre in paired sera, presence of IgM phase II antibodies, or detection of nucleic acid by polymerase chain reaction (PCR) in blood or respiratory tract samples [16]. We excluded 48 cases who were known to have attended farm viewing days at a Q fever positive local petting farm in 2009, which had previously been implicated in transmission of the disease to visitors [17].

### Comparison of 2008 to 2009 cases

We compared the 2009 cases to cases notified in 2008 to the same MHS using descriptive analysis and standard statistics (Student’s T-test or chi-square test as appropriate). Specifically, we examined the cases in terms of time, place and person (epidemic curve, age, gender ratio, and postcode of residence accurate to neighbourhood level), together with hospitalisation rates and time from date of onset to laboratory confirmation to see if there were any significant differences in these parameters in 2009 as compared to 2008. For 2008 cases, we used the same case definition that Schimmer et al. [12] employed in their investigation of the 2008 outbreak. Data were analysed using Stata 12.

### Suspect source farm identification

Van Leuken et al. [14] identified 56 farms holding small ruminants in the area of the 2009 outbreak, of which seventeen were indicated to be potential sources using an exponential model to fit Q fever incidence against increasing distance from each farm. We used three approaches to investigate these seventeen farms further:

1. Collation of veterinary and farm data: we obtained data from the Animal Health Service (GD) regarding the farm location and number of goats and sheep held in 2009 for each of the seventeen farms. We also collated data on Q-fever history of each farm from three sources: (1) clinical Q-fever notifications in 2008 and 2009; (2) results of the non-mandatory bulk tank milk testing for *C. burnetii* in 2008 on large dairy goat farms (for which the take-up rate was ~75 % of dairy goat farms with >50 animals) [18]; (3) results of the mandatory, systematic bulk tank milk testing for *C. burnetii*, introduced in October 2009 and conducted at all dairy farms with >50 goats/sheep [10]; and (4) results of previous animal and environmental Q-fever investigations in the study area [19–21].
2. Concentric ring attack rate analyses: following the approach used by Schimmer et al. [12] to investigate the 2008 outbreak in the area, we used distance of a case’s residence to a farm as a proxy for exposure together with 2009 population data to calculate attack rates (AR) for residents in concentric 1 km rings around each potential source farm, up to a 5 km radius (i.e. 0-1 km, 1-2 km, 2-3 km, 3-4 km and 4-5 km). We also calculated the attack rate in a reference ring of 5-10 km around each potential source farm and used this to compute relative risks (RR) for each 1 km concentric ring. Data were analysed using R version 3.0.3.
3. Mapping: using ArcGIS 9 software (ESRI, Redlands, CA, USA), we geo-referenced the residential addresses of each case according to six figure postcode (discriminatory down to street level) and used 2009 population data to produce smooth incidence maps, as developed by Van der Hoek et al. [22] Briefly, this method imposes a 500 x 500 m grid over the area of interest; counts the number of cases and the population number in each cell; and models the number of cases as an inhomogenous Poisson process whereby the underlying incidence is estimated by 2-dimensional P-spline smoothing. We also mapped the seventeen farms identified as potential sources, together with farm size and animal species. As several farms were in close proximity to each other, the ARs and RRs of a particular farm calculated in the concentric ring analyses could have been an artefact of the *C. burnetii* emissions from a nearby farm; therefore mapping served to visualise whether this phenomenon might be occurring.

### Further investigation of the most strongly suspected farm

We then performed the following additional investigations for the farm most strongly associated with occurrence of cases according to the previous methods (Farm A): (1) further spatio-temporal analyses using this farm as the “emission point” for *C. burnetii*, and (2) an on-site farm visit:

1) Spatio-temporal analyses using an atmospheric dispersion model: we defined a “period of probable infection (PPI)” denoting the period during which a case was likely to have been infected, i.e. 8–33 days (95 % CI) prior to the date of onset of disease [23]. We hypothesised that relatively high modelled concentrations of *C. burnetii* originating from Farm A during the PPI preceding a particular case’s date of onset may indicate that the case in question was infected via airborne transmission from the farm. That is, if a case was infected by exposure to *C. burnetii* from Farm A, it would have been exposed mainly during its PPI rather than during the period prior to that.

To test this hypothesis, we applied an atmospheric dispersion model previously described by Van Leuken et al. [15] to model the airborne transmission of *C. burnetii*. The model calculates the physical dispersion of *C. burnetii* bacteria from a particular emission point (in this case a farm) by taking into account hourly-based meteorological data, including wind velocity and direction. The model output consists of hourly averaged *C. burnetii* concentration matrices which are then converted to period-specific averaged concentration maps.

**Fig. 1:**
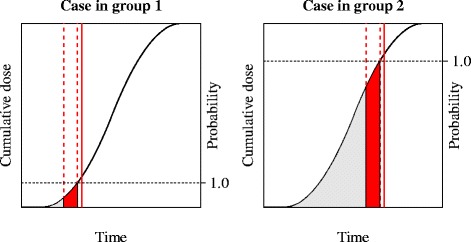
Assignment of cases to group 1 and group 2. Example of cases in groups 1 (left) and 2 (right). The vertical solid red lines indicate a case’s date of onset of disease; the vertical dashed red lines indicate the period of probable infection (PPI), being 8 – 33 days before the date of onset of disease [23]. The grey polygon indicates the cumulative dose prior to the PPI (*C*_PRE_); the red polygon indicates the cumulative dose during the PPI (*C*_PPI_). The probability of infection has increased to 1.0 at the end of the PPI. For cases in group 1 the surface of the red polygon is greater than that of the grey polygon (*C*_PPI_ > *C*_PRE_); and vice versa for cases in group 2 (*C*_PPI_ ≤ *C*_PRE_)

We inserted hourly averaged local meteorological conditions from the Royal Netherlands Meteorological Institute into the model and performed sensitivity analyses on the threshold wind velocity (0, 2, 4 and 6 m/s) and the emission profile: conYear (constant emission in time) or lNormEpi (log-normal emission curve) starting from the first day of the kidding season (for details see Van Leuken et al. [15]). For a case i, we determined the cumulative modelled concentration at its street-level postcode during its PPI (C_PPI,i_), and also prior to the PPI (C_PRE,i_). Subsequently, we divided all cases into two groups: “group 1”, containing all cases who had a higher cumulative concentration during their PPI compared to prior to it (C_PPI_ > C_PRE_); and “group 2”’ containing all cases with a higher cumulative concentration prior to their PPI compared to during the PPI (C_PPI_ ≤ C_PRE_). In other words, a case with a cumulative exposure ratio C_PPI_: C_PRE_ of 90:10 was clearly attributed to group 1, whereas a case with a ratio of 10:90 was attributed to group 2. The cut-off was thus set at 50:50. Figure 1 gives an example of the classification of cases into groups 1 and 2.

We created histograms of both groups as a function of distance from Farm A, and, in addition, we determined the relative abundance of cases (*ϕ*) per group per circular ring r of 1 km:

1 $$ {\phi}_r=\frac{n_{r,1}/{n}_1}{n_{r,2}/{n}_2} $$

where n_r,1_ and *n*_r,2_ are the number of cases in ring *r* in group 1 and 2 respectively, and *n*_1_ and *n*_2_ are the total number of cases per group. Thus, the denominator and the numerator give the proportion of cases in each group as a function of distance. Variable *ϕ* then gives the ratio of these proportions and thus determines which group is over-represented as a function of distance from Farm A.

For the purposes of the model, we assumed that emission of *C. burnetii* from the farm started in week 9 in 2009, i.e. two weeks before the first cases were notified (to correspond approximately with the incubation period of 8 – 33 days [23]). However, to test the dependence of *ϕ* on the week that the emissions commenced, we additionally performed a sensitivity analysis regarding week of emission.

Finally, we performed a Kruskal-Wallis test (a non-parametric test) to compare the median distance of both groups to the suspected source farm (since the cases are spatially clustered and therefore the incidence-distance relationship is exponentially curved [14], these medians (or means) cannot be compared using a standard t-test, which assumes normality). We compared the resulting T-statistic to a χ^2^-distribution with one degree of freedom, assuming a threshold for significance of p < 0.05.

2) Farm visit: on 17 August 2012, we visited Farm A and interviewed the farmer using a semi-structured questionnaire to gain insight into the farm characteristics, husbandry methods, breeding periods, and animal health status in spring-summer 2009.

### Ethics

Q fever is a notifiable disease in the Netherlands and according to Dutch legislation, written consent from patients for the use of anonymised surveillance data is not necessary. Therefore, ethical review was not required. The Municipal Health Service Brabant-Zuidoost made the case information available to the researchers. However, publication of this case information is not permitted.

## Results

### Comparison of 2008 to 2009 cases

**Fig. 2:**
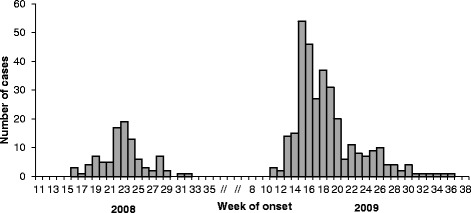
Distribution of Q fever cases over time, 2008 and 2009 outbreaks. The figure shows Q fever cases that were notified to the Brabant-Zuidoost Municipal Health Service during the 2008 and 2009 outbreaks in the area. Cases that were linked to visiting an infected petting farm in 2009 are excluded. [17]

1) Time and Place: a total of 320 cases met the 2009 case definition, compared to 96 in 2008. All cases identified in 2009 were different from those identified in 2008, ie were new cases. Figure 2 shows the distribution of cases over time from 2008 to 2009. The 2008 and 2009 outbreaks both started and ended at broadly similar times, with date of onset of cases between weeks 16 and 32 in 2008 compared to weeks and 11 and 36 in 2009. The peak number of cases occurred in week 23 in 2008 (n = 19) compared to week 15 in 2009 (n = 54). Cases lived in similar postcode areas to the 2008 outbreak [12], with the same two neighbourhoods being most affected both in 2008 and 2009 (65 % of cases and 53 % of cases respectively); these two neighbourhoods are contiguous.

2) Demographics: the age and gender of cases did not differ significantly between 2008 and 2009. In 2008, median age affected was 50, compared to 49 in 2009 (p = 0.79). In 2008, 68.8 % of cases were male compared to 61.6 % in 2009 (p = 0.20).

3) Hospitalisation rate and laboratory confirmation delay: hospitalisation rate in 2009 was higher than in 2008 (23.7 % and 18.8 % respectively), but this difference was not significant (p = 0.56). Laboratory confirmation delay (time between date of symptom onset and date of laboratory confirmation) was significantly shorter in 2009 compared to 2008 (p = 0.0001), with a median delay of 20 days in 2009 compared to 29 days in 2008.

### Suspect source farm identification

#### Collation of veterinary and farm data

Table 1 describes the farms in terms of their size, species and Q fever history. Of the seventeen farms, eight had only goats, seven only sheep and two were mixed. Farm A was the largest farm with 791 goats (no sheep); Farm H was the second largest with 175 sheep (no goats), and the remaining farms all had fewer than 40 animals. Three of the seventeen farms had a history of Q fever. Farm A had clinical Q fever (abortion wave) in 2008, had tested positive for *C. burnetii* during bulk tank milk (BTM) testing in 2008 and 2009; and had almost 100 % of various samples testing positive in previous investigations since the original 2008 outbreak (vaginal and stable environmental swabs in 2009 [20]; and aerosol samples in the stable and the outdoor environment, together with swabs from the stable environment, in 2010 [21]. Farm H had 5/20 positive samples [20], and Farm C had 3/12 positive swabs in 2008 [20]. The remaining fourteen farms were too small (<50 animals) to have qualified for bulk tank milk testing, and had neither been included in other previous animal/environmental investigations; however, none had reported any clinical Q fever nor abortion problems to the Animal Health Service in 2008 or 2009.

**Table 1:** Characteristics and Q fever history of potential source farms

Farm	No. sheep (′09)	No. goats (′09)	Abortion history?	BTM 2008	BTM 2009	De Bruin et al. (2011)	De Bruin et al. (2012)	De Bruin et al. (2013)
A	0	791	April 2008	Positive	Positive	n.i.	48/49 (Oct ′09)	20/20 (May ′09′)
B	0	5	None	n.i.	n.i.	n.i.	n.i.	n.i.
C	0	8	None	n.i.	n.i.	3/12 (′08)	n.i.	n.i.
D	37	0	None	n.i.	n.i.	n.i.	n.i.	n.i.
E	2	0	None	n.i.	n.i.	n.i.	n.i.	n.i.
F	31	0	None	n.i.	n.i.	n.i.	n.i.	n.i.
G	0	2	None	n.i.	n.i.	n.i.	n.i.	n.i.
H	175	0	None	n.i.	n.i.	n.i.	n.i.	5/20 (May ′09)
I	36	0	None	n.i.	n.i.	n.i.	n.i.	n.i.
J	0	2	None	n.i.	n.i.	n.i.	n.i.	n.i.
K	9	0	None	n.i.	n.i.	n.i.	n.i.	n.i.
L	0	2	None	n.i.	n.i.	n.i.	n.i.	n.i.
M	4	3	None	n.i.	n.i.	n.i.	n.i.	n.i.
N	4	4	None	n.i.	n.i.	n.i.	n.i.	n.i.
O	0	6	None	n.i.	n.i.	n.i.	n.i.	n.i.
P	9	0	None	n.i.	n.i.	n.i.	n.i.	n.i.
Q	0	2	None	n.i.	n.i.	n.i.	n.i.	n.i.

Farm characteristics (number of sheep and goats in 2009) and known Q fever history (abortion, bulk tank milk screening, and results of previous targeted Q fever studies) for the seventeen potential source farms, Note that bulk tank milk (BTM) was monitored by the Animal Health Service on a volunteer basis from October 2008 at dairy farms with >50 goats/sheep [18], and was mandatory from October 2009 for all dairy goat and sheep farms with >50 animals [10]. For the previous Q fever studies [19–21], we have indicated the number of positive vaginal and/or stable environmental samples found;. “n.i.” means that the farm was not included in that particular study

#### Concentric ring attack rate analyses

**Fig. 3:**
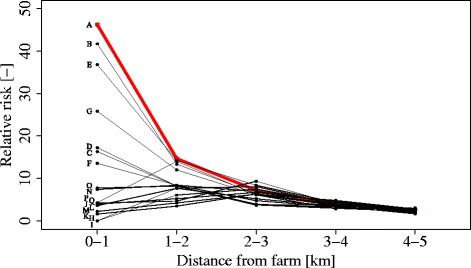
Relative risks per concentric ring for the seventeen selected farms A – Q. The curve of Farm A is displayed in red

Figure 3 shows the Relative Risk for Q fever with increasing distance from each of the seventeen farms (data are presented in Additional file 1: Table S1). Farms A, B and E had the most striking distance-response relationship, followed by G, and then D, C, and F to a lesser extent. The remaining ten farms showed no clear relationship between distance from farm and RR. Farm A had the highest overall attack rate in the 0–1 km ring at 2156 cases per 100,000 population, although the 0-1 km attack rates for Farms B (1802 cases per 100,000 population), E (1818 cases per 100,000 population), F (1031 cases per 100,000 population) and G (1132 cases per 100,000 population) were not statistically significantly different to this figure. Farm A also had the highest relative risk in the 0–1 km ring compared to the 5–10 km reference ring (RR = 46; 95 % CIs 22–97).

#### Mapping

**Fig. 4:**
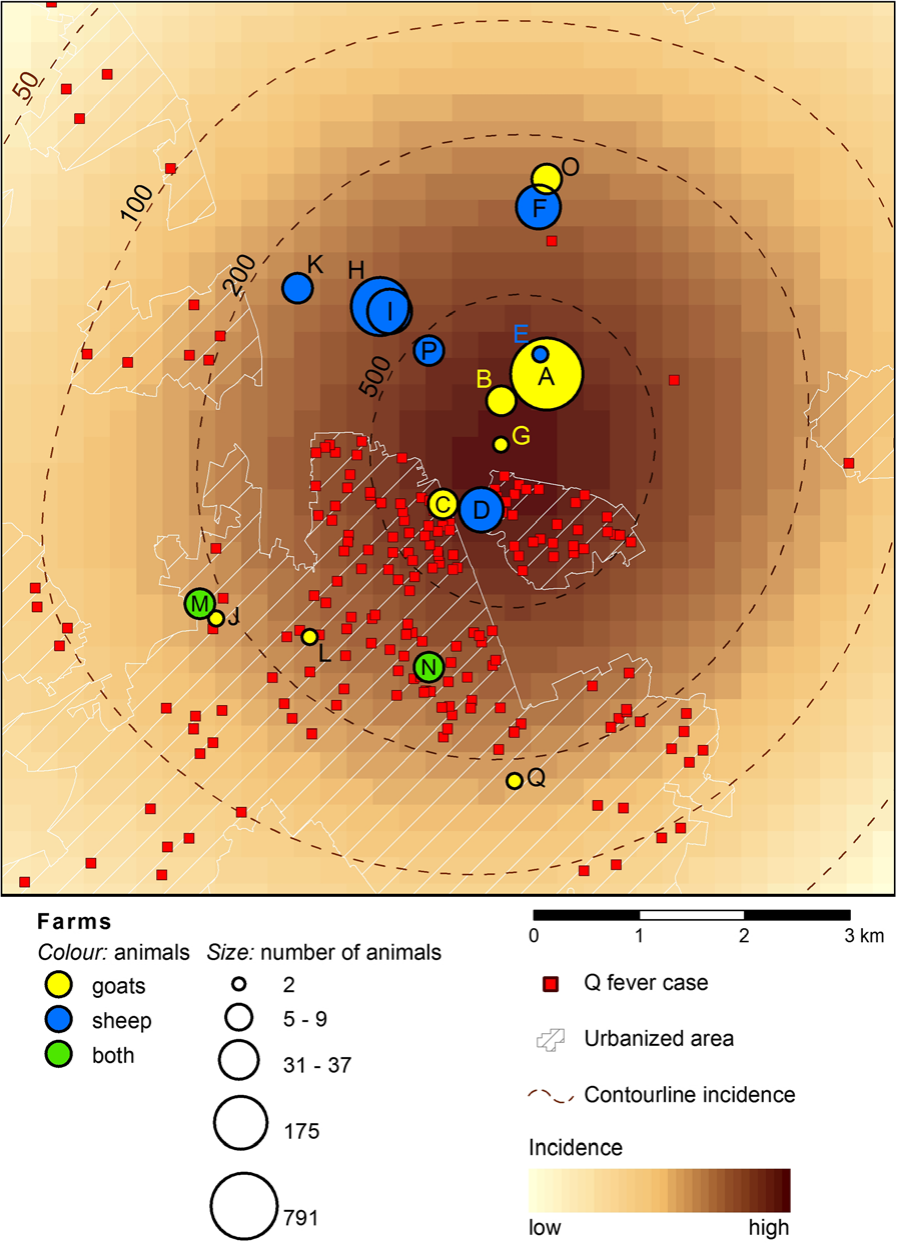
Map of the residential locations of cases (six-digit postcode) and the location of the seventeen farms A – Q selected from the study of van Leuken et al. [14] with the size of the circle proportional to their number of animals (yellow: goat farms, blue: sheep farms, green: farms that kept both goats and sheep). As a background the incidence is presented as a smoothed map, based on the method of van der Hoek et al. [22]

Figure 4 shows the map of the residential addresses of notified cases, the location and size of the seventeen potential source farms, and the smooth incidence of human cases in the locality over the outbreak period (weeks 11–36). Reflecting the results of the concentric rings analysis, Farms B and E were in closest proximity to Farm A (502 m and 196 m respectively), followed by Farm G (801 m).

#### Further investigation of the most strongly suspected farm

Taking all the above information together, Farm A was identified as the most strongly suspected potential source farm. Our reasoning for this is as follows: it was by far the largest farm of the seventeen; it had a confirmed history of clinical Q fever (2008) plus positive BTM investigations subsequently in 2008 and 2009; it was a goat farm rather than a sheep farm (goat farms being more frequently indicated in the Dutch Q fever epidemic than sheep farms [11]); and the results of the concentric rings analysis combined with the mapping pointed most strongly towards it (although Farms B and E also had very strong distance-RR relationships in the concentric rings analysis, the mapping showed that they were both very close to Farm A. Therefore we considered that these relationships were likely to be an artefact of proximity, particularly given that Farm B held only five goats and Farm E, two sheep).

#### Spatio-temporal analyses using an atmospheric dispersion model

**Fig. 5:**
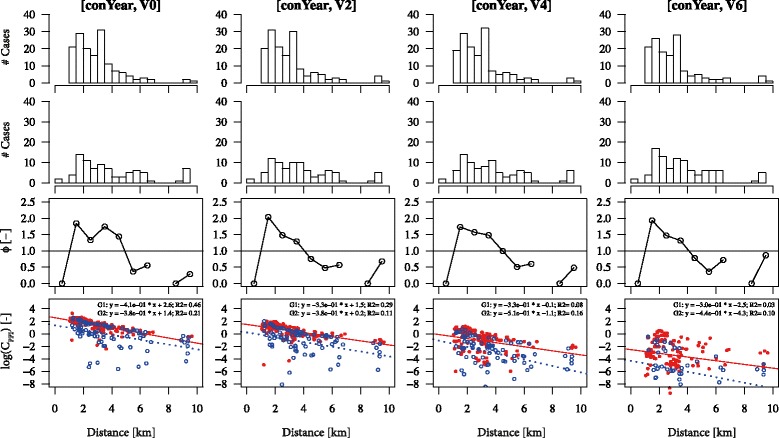
Results of spatio-temporal analysis. Plots as function of the threshold wind velocity (V0, V2, V4, V6) for emission profile *conYear.* Upper row: histograms of the number of cases in group 1 as function of distance [km]. Second row: idem for group 2. Third row: ratio of the proportion of cases (*ϕ*) as function of distance per 1 km. Fourth row: logs of the cumulative concentrations during the incubation period (C_PPI_) per group (G1 [red bullet], G2 [blue bullet]) as a function of distance. Equations of the linear regression lines are given as well as the R^2^’s of the data

Figure 5 (upper two rows) shows the histograms of the number of cases in groups 1 and 2 as a function of distance for emission profile *conYear*, stratified by the threshold wind velocity (see Additional file 2: Figure S1 for emission profile *lNormEpi*). These results show that approximately 60 – 65 % of the cases are attributed to group 1. Also, the emission profile and threshold wind speed have little influence on the shape of the distributions. The third row shows the ratio of the proportions (*ϕ*) from equation 1: group 1 is over-represented at distances up to 5 km (i.e. the points lie above 1.0). At distances of 5 – 7 km, in contrast, group 2 seems to be over-represented (i.e. the points lie below 1.0), although the absolute number of cases is smaller. Note that the first point should be interpreted carefully as the number of cases up to 1000 m in group 1 is equal to zero. Additional file 3: Figure S2 shows the actual cumulative concentrations (C_PPI_ and C_PRI_) per case.

**Fig. 6:**
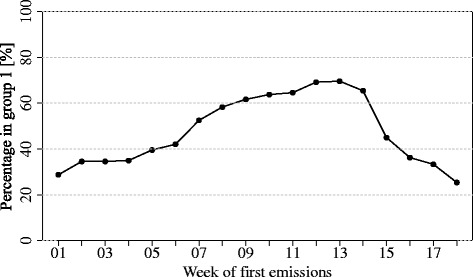
Sensitivity analysis. Sensitivity analysis demonstrating the percentage of cases in group 1 as a function of the week number that the emissions from Farm A would have started

Figure 6 shows the results of the sensitivity analysis of the number of cases in group 1 as a function of the starting week of emissions (assuming emission profile *conYear* and a threshold wind velocity of 2 m/s). In our model, we assumed that the emissions commenced in week 9, corresponding with about 61 % of the cases classified to group 1; however, if we had set the first week of emissions to be week 12 or 13, then this number would have been slightly higher at approximately 69 %.

Table 2 shows the median distances of a case’s residence to Farm A per group as a function of the threshold wind velocity. The 95 % confidence intervals were determined by bootstrapping 10,000 times. According to the Kruskal-Wallis test the median distance of group 1 is always significantly lower (p ≈ 0.004) than that of group 2, indicating that cases of group 1 are, on average, living closer to Farm A than cases of group 2. The fraction of cases classified to group 1 has little association with emission profile or threshold wind velocity.

**Table 2:** Median distances of cases to Farm A per group as function of the threshold wind velocity

Emission profile	Threshold wind speed	Number of cases		Distance to Farm A (median and 95 % CI)		Kruskal-Wallis *p*-value
		Group 1	Group 2	Group 1	Group 2	
con Year	0	148	92	2760 [2345; 3062]	3207 [2680; 3652]	0.0094
	2	148	92	2612 [2182; 2970]	3491 [3002; 3890]	0.0001
	4	154	86	2735 [2326; 3038]	3308 [2804; 3692]	0.0055
	6	144	96	2694 [2372; 3018]	3352 [2968; 3742]	0.0054
lNormEpi	0	155	85	2747 [2372; 3053]	3259 [2804; 3789]	0.0029
	2	153	87	2661 [2222; 3018]	3393 [2982; 3751]	0.0012
	4	151	89	2715 [2301; 3022]	3372 [2812; 3742]	0.0032
	6	140	100	2670 [2274; 3018]	3317 [2920; 3602]	0.0026

Number of cases and median distance of cases’ residence (six-figure postcode) from Farm A per group, stratified by emission profile and the threshold wind speed. The last column gives the p-values of the Kruskal-Wallis test

Finally, the bottom row in Fig. 5 shows the logs of the cumulative concentrations that cases were exposed to during their PPI (C_PPI_) for emission profile *conYear* and threshold wind velocities of 0, 2, 4, and 6 m/s (V0, V2, V4, V6 respectively). The results show that the differences between the groups were very small, but for higher threshold wind speeds the cumulative concentration during the PPI was higher in group 1. Note that the data become more scattered for high threshold wind speeds: this is caused by a more plume-shaped exposure pattern (with higher contrast) for the cumulative concentration maps (see Additional file 3: Figure S2).

Visit to Farm A: in 2009, the goats were kept on a deep litter system with fresh straw added daily. The shed was mechanically ventilated. The litter was removed three to four times during the year. No further cleaning was done before replenishing with fresh litter. Birthing products were not cleared from the pens during the kidding season. The farmer could not recall any particular changes in management in the spring/summer of 2009, nor any animal health problems around the time of parturition or at any other time during the year. The goats had not been vaccinated against Q fever in 2008, but were vaccinated against the disease in May/June 2009. Although the farmer had collected data regarding the period of kidding, numbers of kids born and number of live/still births in 2009 for submission to the Animal Health Service, he no longer had these data available. We were unable to access these data from the Animal Health Service independently.

## Discussion

This study aimed to identify the most likely origin of an outbreak of Q fever in an urban area in 2009 that occurred in the absence of any clinical Q-fever notifications from surrounding farms that year. Our results suggest that the most likely source of this outbreak was the same dairy goat farm, Farm A, as had been implicated in a smaller outbreak in the area the previous year. We gathered data from a wide range of sources and sectors (animal, human, environmental and meteorological) and applied a range of interdisciplinary methods incorporating both traditional and innovative techniques in order to reach this conclusion. Integrated approaches such as these are increasingly recognised as key to One Health research. The mechanisms underlying zoonotic disease emergence and transmission are invariably complex and multi-factorial, such that understanding them requires interdisciplinary work that applies a holistic set of methodologies and data from the human health, veterinary, and environmental sectors [24, 25]. For the purposes of this study, we also developed a new method that enabled the integration of spatial data generated through an atmospheric dispersion model with temporal data concerning time of probable infection. This method could prove valuable to future Q fever outbreak investigations as it can serve to disentangle acute aerosol transmission from a point source from diffuse environmental transmission.

While comparison of the area’s 2008 outbreak data to its 2009 outbreak data suggests that there was a higher awareness of Q fever among the public and medical community in the area in 2009 compared to 2008 (demonstrated by the significantly shorter period between date of onset of disease and date of laboratory confirmation in 2009), the similar demographics of cases in terms of time, place and person and the lack of a reduction in hospitalisation rate do not support the hypothesis that the increase in cases of Q fever seen in 2009 was caused solely by changes in reporting behaviour. Instead, our results suggest that this was a true outbreak rather than a reporting artefact, with Farm A as the probable source. The timing of the 2009 outbreak (in the spring around the kidding season) suggests that the outbreak was due to goats sub-clinically shedding *C. burnetii* during this period, rather than due to environmental contamination of Farm A and its surrounds during 2008.

This is supported by the results of spatio-temporal analyses using an atmospheric distribution model. By means of this method, we calculated the proportion of cases that were mainly exposed during their probable period of infection (PPI), namely 60 – 65 % of the cases. Thus, these cases could be explained by airborne transmission of *C. burnetii* from Farm A. The other cases were mainly exposed prior to their PPI; explanations for this could be that (1) they were absent from home during high-exposure hours/days, or (2) they did not happen to inhale an infected particle during the PPI by chance, despite high concentrations around their area of residence at that time, or (3) they were infected by a different source (including re-aerosolised bacteria from contaminated environments).

It has been suggested that *C. burnetii* may still be excreted via the placenta during normal parturition [3, 4, 26, 27] even in vaccinated animals, albeit to a lesser extent [28]. A recent meta-analysis of studies investigating the effect of vaccination on shedding suggested that while vaccination significantly reduced the risk of shedding form uterine secretions in previously exposed goats, shedding through all other routes (milk, vaginal secretions and faeces) was not significantly reduced. [29] Furthermore, farms that have experienced Q fever-related abortions in the past have been shown to have higher proportions of *C. burnetii*-positive animal and environmental samples, and higher levels of *C. burnetii* DNA within those positive samples compared to both farms negative for Q fever and farms positive on bulk milk sampling but with no history of abortion. Unfortunately detailed veterinary/husbandry data were unavailable regarding the start, end and peak kidding periods or other on-farm events in 2009 (e.g. removal of litter from the pen) that might have contributed to the rise in human cases. For the purposes of the model, we assumed that *C. burnetti* emission started in week 9, two weeks before the first notification. Although the proportion of explained cases would have been larger had we assumed an emission start in week 12 or 13 (Fig. 6), cases with weeks of onset between weeks 11 and 14 would subsequently have been missed by the model.

The model also demonstrated a strong relationship between distance from cases’ residence to Farm A and incidence of Q fever stratified by group. Cases belonging to group 1 (i.e. mainly exposed during their PPI) were over-represented at distances of up to 4 to 5 kilometres from Farm A, while cases in group 2 were living at relatively further distances of 5 to 8 kilometres (there were too few cases at 9 km and 10 km to draw conclusions about these distances). Also, cases in group 1 were generally exposed to higher doses during their PPI (Fig. 5) compared to cases in group 2. Note that we did not look at the difference in absolute exposure between cases, but instead at the exposure per case. After all, the probability of infection is always 100 % at the end of a case’s PPI, regardless of the actual dose. Nevertheless, other sources could have increased the actual dose and thus the probability of infection. Furthermore, while choice of emission profile (i.e. *conYear* and *lNormEpi)* and threshold wind velocity had only a minor effect on the proportion of explained cases (Fig. 5 and Additional file 2: Figure S1), exposure at short distances up to 4-5 km was an important indicator for disease. These results complement the findings from concentric rings attack rate analyses in both this study and previous studies [12, 13] which have indicated that persons living within 5 km of an infected farm are at particular risk of contracting Q fever.

Although seventeen farms were included for investigation at the outset, we focussed our farm visit and spatio-temporal analyses to include Farm A only. It could be argued that, despite the weight of evidence pointing to Farm A, similar investigations should have been performed at all farms for the purposes of fair comparison. One argument for focussing on Farm A was its size; with 791 animals it was by far the largest farm of the seventeen: indeed, fifteen farms had under 40 animals, twelve of which had under ten. The only other large farm, Farm H (175 sheep), was not further investigated because the concentric rings analysis and smooth incidence mapping did not point towards it; however, five out of 20 environmental and aerosol samples taken at and around the farm in 2009/2010 tested positive for *C. burnetii* [21]*.* Furthermore, although it may be considered unlikely that the small farms would be the source of such a large human outbreak, it is theoretically possible. Unfortunately, because these farms were so small, they did not fall under statutory regulations for bulk tank milk sampling; neither had they been included in previous investigations, so we had no data to rule out Q fever in these farm animals. Ideally, all seventeen farms would have been individually visited and animals sampled as part of our investigations; however, our resources did not permit this and we focussed on the most strongly suspected farm only. With regards the spatio-temporal modelling: the concentric rings analyses suggested most strongly that farms A, B and E, and possibly G, warranted further investigation. However, as these farms were so close to Farm A, any results yielded from atmospheric dispersion model centred on these farms would have been almost identical to that for Farm A and therefore would have yielded almost identical information.

Caution is called for in the interpretation of the spatio-temporal analyses. The method requires that cases are assigned into one of two distinct groups: those who were exposed to a higher modelled concentration of *C. burnetii* in the 8–33 days before date of onset (the period of probable infection, PPI) compared to prior to it (group 1), and those who had a higher modelled concentration prior to their PPI compared to during the it (group 2). However, this was not always a straightforward distinction (Fig. 5) and some cases may have been mis-assigned. It is difficult to extrapolate from modelled concentration at a particular postcode area to the actual level of exposure of a person in reality: for example, a case may have been absent from their residence during their PPI; there may have been a relatively low number of contaminated particles in their particular street; or they may have been exposed to other sources in addition to Farm A.

## Conclusions

Our study found that most likely source of a large urban outbreak of Q fever in 2009 was the same dairy goat farm implicated in a smaller outbreak in the area the previous year. The results of our investigations highlight that farms with a history of Q fever can still pose a risk to public health even when there are no animal health indicators such as abortion storms or other fertility problems. We recommend that veterinary and public health professionals consider farms with past as well as current history of Q fever as potential sources of human outbreaks. We used a variety of methods to reach our conclusions, including a newly developed method to investigate such outbreaks in space and time using an atmospheric dispersion model. Using meteorological forecast data, this method could potentially be used prospectively to visualise the spread of *Coxiella* bacteria and inform infectious disease prevention and response measures.

## Additional files

Additional file 1: Table S1. Attack rates and relative risks per potential source farm. Number of cases, number of total inhabitants, attack rates (AR) per 100,000 inhabitants, and relative risks (RR) per concentric 1 km ring around each potential source farm A – Q. (XLS 29 kb) Additional file 2: Figure S1. Results of spatio-temporal analysis using an alternative emission profile. As for Fig. 5, but for emission profile *lNormEpi. (PDF 564 kb)*
Additional file 3: Figure S2. Actual cumulative concentrations (C_PPI_ and C_PRI_) per case. Cumulative concentration during the PPI (*C*_PPI_) as a function of the cumulative concentration prior to the PPI (*C*_PRE_) for each case (emission profile *conYear*, threshold wind velocity 2 m/s). Cases in group 1 are denoted by red closed circles [red bullet]; cases in group 2 by blue open circles [blue bullet]. (PDF 7 kb)

## Abbreviations

AR: Attack Rate
BTM: Bulk Tank Milk
GD: Animal Health Service
GIS: Geographic Information System
MHS: Municipal Health Service
PPI: Period of Probable Infection
PCR: Polymerase Chain Reaction
RR: Relative Risk
