# Supplementary figures and images for: Integrating interdisciplinary methodologies for One Health: goat farm re-implicated as the probable source of an urban Q fever outbreak, the Netherlands, 2009
Source: BMC Infect Dis. 2015 Sep 3;15:372. doi: 10.1186/s12879-015-1083-9 (PMC4558730; doi:10.1186/s12879-015-1083-9)

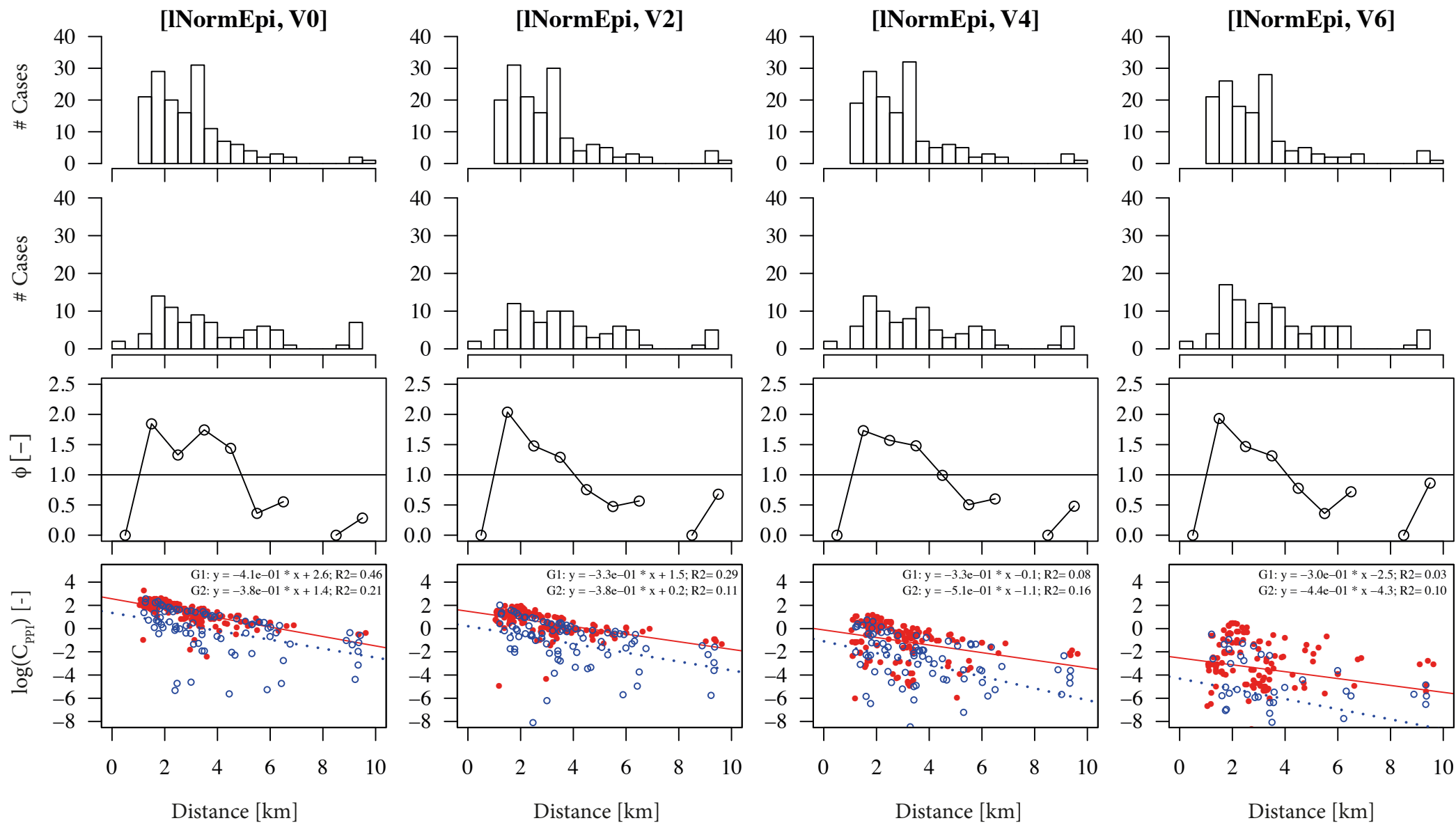

Supplement: Additional file 2: Figure S1. — Results of spatio-temporal analysis using an alternative emission profile. As for Fig. 5, but for emission profile lNormEpi. (PDF 564 kb) [file 12879_2015_1083_MOESM2_ESM.pdf]

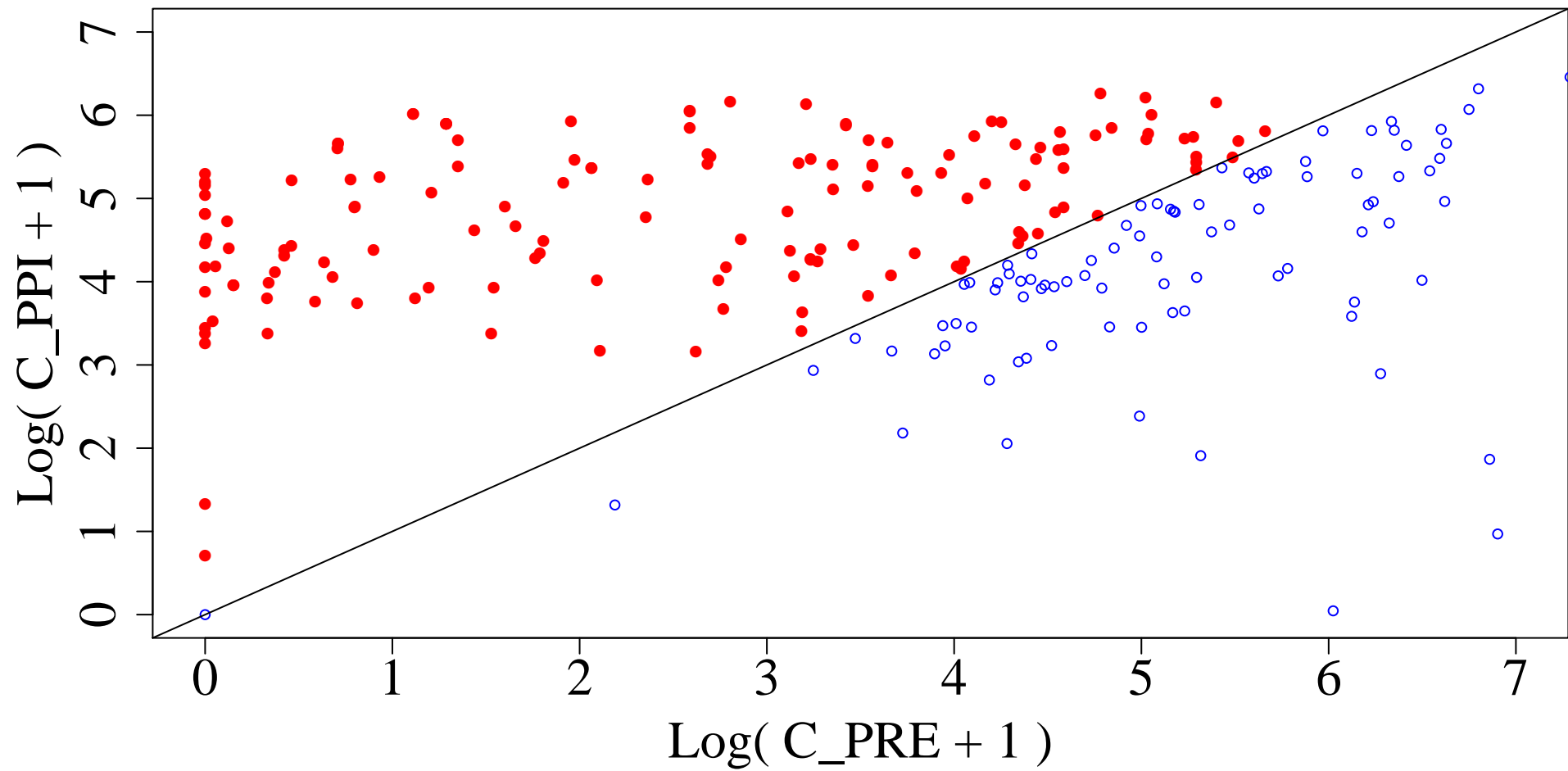

Supplement: Additional file 3: Figure S2. — Actual cumulative concentrations (CPPI and CPRI) per case. Cumulative concentration during the PPI (C PPI) as a function of the cumulative concentration prior to the PPI (C PRE) for each case (emission profile conYear, threshold wind velocity 2 m/s). Cases in group 1 are denoted by red closed circles [red bullet]; cases in group 2 by blue open circles [blue bullet]. (PDF 7 kb) [file 12879_2015_1083_MOESM3_ESM.pdf]
